# Association of Ohio C. D. S.

**Published:** 1852-04

**Authors:** 


### ASSOCIATION OF THE OHIO COLLEGE OF DENTAL SURGERY.

At a meeting of the stockholders of the Ohio College of Dental Surgeons, held in the College Hall in Cincinnati, at 10 o'clock A. M. of the 19th February, 1852, Dr. Chas. Bonsall was called to the chair, and Dr. Thos. Wood appointed Secretary.

On motion, it was resolved that the chair appoint a committee of three to draft a constitution for the government of this association. The chair thereupon named the following gentlemen: Drs. Thos. Wood, H. R. Smith and James Taylor.

After considerable expression of opinion, which was asked for by the committee, adjourned to meet at the house of Dr. James Taylor, at 7 o'clock P. M.

### EVENING SESSION.

Met according to adjournment, and after quite an interesting discussion of creature comforts, the constitutional committee reported the form of a constitution, which was considered by sections, and after some modifications, the following was adopted.

### PREAMBLE.

The stockholders and alumni of the Ohio College of Dental Surgery believing that the interests of dental science require a a more thorough course of dental instruction than has heretofore usually been afforded, and that this can best be accomplished by institutions devoted expressly to this object, and that associations entered into with the proper spirit must afford increased facilities for our mutual improvement, and for the promotion of dental science, and that to further the views of those who have already engaged in the enterprize of permanently founding the Ohio College of Dental Surgery; therefore, for the promotion of these objects, and all such others as may conduce to the advance of our science, we adopt the following

